# Transcriptome Integration Analysis at Different Embryonic Ages Reveals Key lncRNAs and mRNAs for Chicken Skeletal Muscle

**DOI:** 10.3389/fvets.2022.908255

**Published:** 2022-06-16

**Authors:** Pengfei Wu, Kaizhi Zhou, Jin Zhang, Xuanze Ling, Xinchao Zhang, Peifeng Li, Li Zhang, Qingyu Wei, Tao Zhang, Kaizhou Xie, Genxi Zhang

**Affiliations:** ^1^College of Animal Science and Technology, Yangzhou University, Yangzhou, China; ^2^College of Animal Science, Shanxi Agricultural University, Taiyuan, China

**Keywords:** skeletal muscle, lncRNAs, mRNAs, Bian chicken, growth and development

## Abstract

The growth and development of skeletal muscle at embryonic stages are vital and it directly affects the growth performance of chickens. Long non-coding RNA (lncRNA) plays an important role in this process. In the experiment, we collected the leg muscles of fast- and slow-growing Bian chickens both at 14- and 20-day embryo ages (14E and 20E) for RNA-seq. Finally, 292 and 347 differentially expressed (DE) lncRNAs were identified in F14vsF20 and S14vsS20, and 1,295 and 1,560 DE mRNAs were also screened, respectively. Then we constructed lncRNA-mRNA networks for the two groups, respectively, and found that 6 of the top 10 lncRNAs ranked with degree are same. GO analysis showed that 12 of the top 20 terms were same in the two comparison groups and most of them were related to energy metabolisms, such as cellular respiration and aerobic respiration. KEGG enrichment revealed that up to 16 pathways of the top 20 in F14vsF20 were same as that of S14vsS20 and most of them were related to growth, including citrate cycle (TCA cycle) and oxidative phosphorylation. Further analysis showed that there were 602 and 102 same DE mRNAs and DE lncRNAs between the two comparison groups. We then identified 442 lncRNA-mRNA pairs, including 201 mRNAs and 32 lncRNAs. Protein-Protein Interactions (PPI) network was predicted for the 201 mRNAs and three core networks were obtained using the plug-in MCODE of Cytoscape. Then the function of genes in the three core networks was further analyzed with ClueGo and they were mainly enriched in six groups of biological processes. On this basis, combined with KEGG pathways and lncRNA-mRNA networks, we identified several candidate lncRNAs and mRNAs. Among them, lncRNAs mainly include TCONS_00061389, TCONS_00025495, TCONS_00017622, TCONS_00216258 and TCONS_00084223, and mRNAs include PLK1, BUB1, TTK, NDUFS7 NDUFAB1, PDHA1, CDK1, SDHA, ACO2 and MDH1. The results would provide a foundation for further experiments on the role of lncRNAs in the regulation of muscle development. And it could also contribute to further clarify the regulatory mechanism of chicken skeletal muscle.

## Introduction

In recent years, consumer demand for meat with low fat and high protein levels has led to a significant increase in the consumption of poultry meat (1). The skeletal muscle of chicken directly determined the meat production for the broiler industry (2). Studies have shown that the total skeletal muscle fiber number is determined during embryogenesis (3–5). Postnatal growth of skeletal muscle is mainly realized through increases in length and girth of the myofibers, but not by an increase in the number of muscle fibers (6). Therefore, the growth and development of embryonic skeletal muscle are of great significance.

Studies have found that skeletal muscle development is regulated by a variety of genes, such as MSTN, PAXs, MRFs, MEF2, TGF-β and MyHC, etc. However, according to statistics, 75% of mammalian genomes are transcribed, only 2% of which encode proteins, and the rest are all non-coding RNAs (7, 8). Long non-coding RNA (lncRNA), as one of the main members of non-coding RNAs, has been found to play an important role in skeletal muscle growth and development in the past few years (9).

LncRNA H19 is one of the highly expressed lncRNAs identified earlier in animal skeletal muscle (10). Initially, H19 was found to control reactivation of the imprinted gene network during muscle regeneration in mice (11). Studies in porcine satellite cells (PSCs) also revealed that H19 regulated PSCs differentiation through two different pathways (12). On the one hand, H19 functions as a molecular sponge of miR-140-5p, which inhibited the differentiation of PSCs. On the other hand, H19 regulated PSCs differentiation through directly binding with DBN1. Yong et al. (13) found that lncRNA MALAT1 could accelerate skeletal muscle cell apoptosis by recruiting EZH2 in the mouse. Schutt et al. (14) described that the linc-MYH could regulate muscle stem cell numbers and skeletal muscle hypertrophy by affecting the composition of the INO80 chromatin remodeler complex in mice. In the study of chicken skeletal muscle, it was found that lncRNA-six1 could activate the gene Six1 in cis-acting by encoding a micro peptide of about 7.26 kDa and promoting the proliferation and differentiation of chicken myoblasts (15). In addition, lncRNA-six1 could also affect the proliferation and differentiation of myoblasts as a ceRNA by adsorbing miR-1611 (16).

In model animals, the continuous development of high-throughput sequencing and new molecular biotechnology has greatly promoted the research of skeletal muscle-related lncRNA (17, 18). However, lncRNA-related studies are relatively less in the field of agricultural animals, especially on skeletal muscle of chicken in embryonic stages. Until now, only two reports (19, 20) on screening differential lncRNAs of skeletal muscle during chicken embryonic development by RNA-seq technology has been retrieved. Therefore, the identification of major lncRNAs related to chicken skeletal muscle growth and development in embryonic stages will provide a necessary theoretical basis for the genetic improvement of growth traits in chicken.

Bian chicken is an eminent Chinese native breed, which is characterized by its adaptability to poor quality feeds and cold (21). However, the growth rate of Bian chicken is slow, which seriously limits its industrialization. Age of 14-day embryo is the key period for myoblasts in skeletal muscle to proliferate and differentiate into myotubes and finally fuse into muscle fibers, which have been basically fixed at 20-day embryo age. In the study, we collected leg muscles of 14- and 20-day embryos of Bian chickens for RNA-seq to explore the key lncRNAs and their regulatory mechanism of skeletal muscle during embryonic development. This experiment will provide a basis for the further research and also lay the foundation for enriching the mechanisms of lncRNA regulating skeletal muscle growth and development of chicken.

## Materials and Methods

### Ethics Statement

The animal experiments performed in the study were all evaluated and approved by the Animal Ethics Committee of Yangzhou University.

### Animals and Tissues

**Figure 1:**
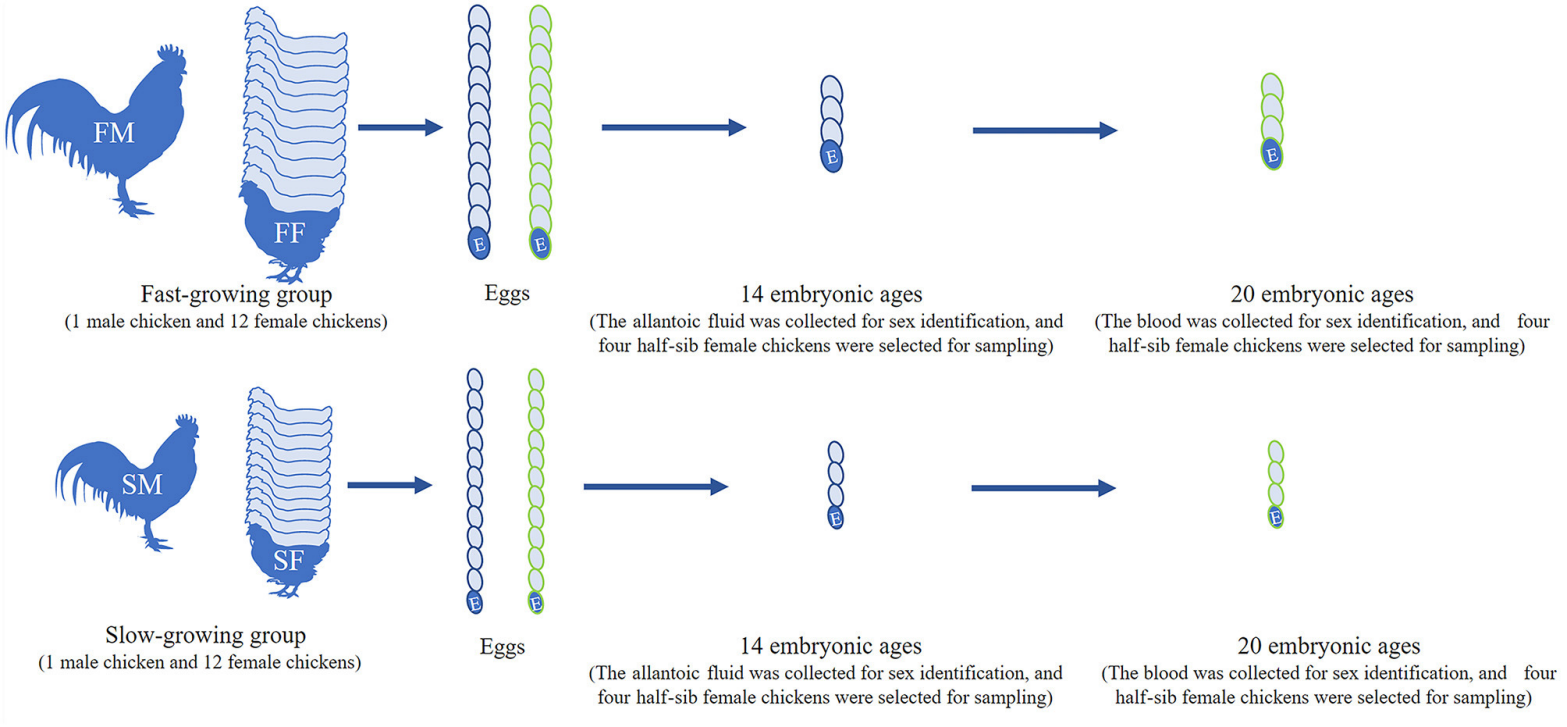
Workflow of sample selection. FM, fast-growing male chicken; FF, fast-growing female chicken; SM, slow-growing male chicken; SF, slow-growing female chicken.

Bian chicken is an eminent native Chinese breed. Our team has established the slow-growing and fast-growing groups with the gene-assisted selection for growth traits, and the chicken has been bred to the seventh generation. At the age of 300 days, twelve female and one male Bian chickens closing to the average weight were selected from the slow-growing Bian chicken group. After artificial insemination, the fertilized eggs were collected and hatched with a temperature of 37°C and humidity of 60%. Do the same work on the fast-growing Bian chicken group as on the slow-growing chickens. Hatching to 14- and 20-day embryo ages (14E and 20E), the eggshell was destroyed and the chick embryos were decapitated rapidly. At the same time, a small amount of allantoic fluid at 14E and blood at 20E were collected for sex identification. Then the left leg muscles were collected and frozen in liquid nitrogen immediately and the sampling process is shown in Figure 1.

**Figure 2:**
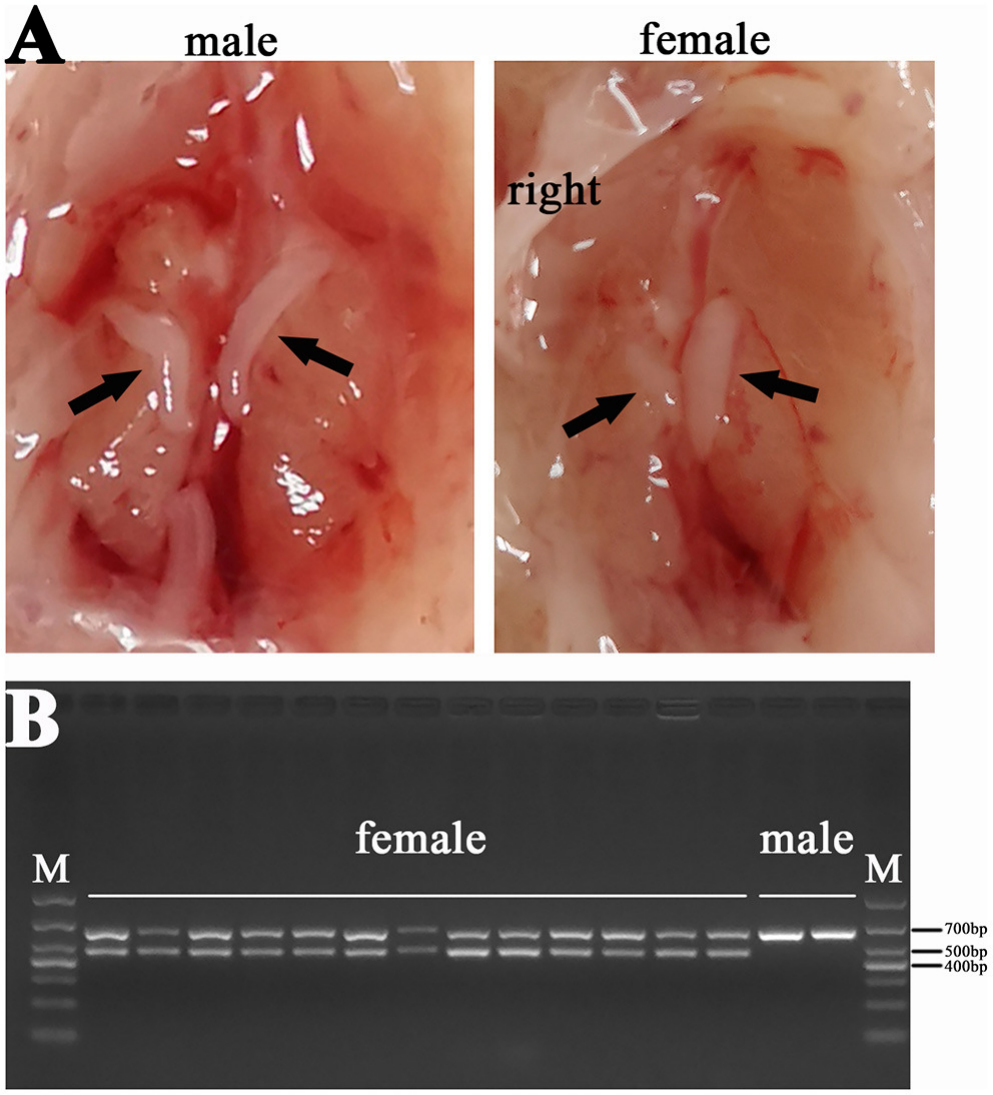
Sex identification results of chicken **(A)** Comparison between male and female gonads: the gonads on both sides of the male chicken develop normally, and the gonads on the right side of the female chicken degenerate; **(B)** Results of PCR for CHD1 gene: two bands were female chicken (ZW) and one band was male chicken (ZZ).

We also dissected the chicken embryos and preliminarily determined their gender by gonadal observation because the right gonad of hens would degenerate (Figure 2A). The CHD1 gene was amplified using allantoic fluid or the full blood to further identify the sex of chicken embryos (Figure 2B) and the the primer sequences are as follows: F: GTTACTGATTCGTCTACGAGA, R: ATTGAAATGATCCAGTGCTTG. PCR reaction was performed with 2xTaq Plus MasterMix (CW2849, CoWin Biosciences, Jiangsu, China) according to the instructions. Template DNA is replaced by allantoic fluid/the full blood with 4 μl/0.5 μl in each reaction. The number of cycles in the reaction process is 35 and the annealing temperature is 55°C. Finally, the left leg muscles of four female embryos with half-sib relationship in each group were used for RNA-seq.

### The Construction of cDNA Library and Sequencing

Total RNA was extracted with TRIzol reagent. The cDNA libraries were constructed with the method of strand-specific sequencing library (22). Briefly, the ribosomal RNA was depleted from total RNA samples using the rRNA Removal Kit following the manufacturer's instruction and then they were broken into 250–300 bp fragments. The first-strand cDNA was reverse-transcribed using fragmented RNA and dNTPs and the second-strand cDNA was synthesized using DNA polymerase I and dNTPs. After adenylation of 3' ends of DNA fragments, sequencing adaptors were ligated to the cDNA. To select cDNA fragments of preferentially 250–300 bp in length, the library fragments were purified with the AMPure XP system. Uridine digestion was performed using Uracil-N-Glycosylase, which was followed by the cDNA amplification using PCR for the construction of cDNA libraries.

The qualified cDNA libraries were finally sequenced on the Illumina NovaSeq 6000 platform and 150 bp paired-end reads were generated.

### Sequencing Data Analysis

Raw data (raw reads) of FASTQ format were processed through in-house Perl scripts developed by Novogene (Beijing, China). In this step, clean data (clean reads) were obtained by removing reads containing adapter, reads containing ploy-N and low-quality reads (more than 50% nucleotides with Q20). At the same time, Q20, Q30, and GC content of the clean data were calculated. All the downstream analyses were based on clean data with high quality.

Clean reads for each sample were then mapped to the reference genome GRCg6a with the software HISAT2 (v2.0.5) (23). Reads alignment results were transferred to the program StringTie (v1.3.3) for transcript assembly (24). All the transcripts were merged using Cuffmerge (v2.2.1) software. Novel transcripts were then obtained from the assembled transcripts with the software Cuffcompare (v2.2.1). The prediction of protein-coding capability for the novel transcripts was performed using CNCI (-m ve -p 1), Pfam (V1.3, -pfamb) and CPC2 (V3.2.0, -pfamB). Among them, transcripts without coding potential in the three software were identified as novel lncRNA and transcripts with coding potential in all the three software were identified as novel mRNA.

### Differential Expression Analysis

Quantification of the transcripts was performed using StringTie (v1.3.3) software and EdgeR (3.2.4) was used for differential expression analysis. Finally, transcripts with P_adjust ≤ 0.05 were assigned as differentially expressed.

### Functional Enrichment

Target gene prediction of lncRNAs was carried out in two ways: co-location and co-expression. Based on the theory of co-location regulation, the protein-coding genes located within 10/100 kb from lncRNA were selected as potential targets (25). While for co-expression target prediction, Pearson's correlations coefficients between the genes and lncRNAs were calculated and were used for the identification of co-expression regulatory relationship (26). Finally, the Gene Ontology (GO) and KEGG enrichment analysis was implemented by the GOseq (R-3.3.2) and KOBAS (v2.0), respectively (27, 28).

### Construction of Candidate Gene Network and Theirs Function Analysis

Protein-Protein Interactions (PPI) analysis of DE mRNAs was based on the STRING database (29) and Cytoscape (3.8.2) was used to visualize the PPI networks. Finally, we used the plug-in MCODE to obtain the core networks for PPI networks and the plug-in ClueGO was further used to analyze the biological functions of genes in each core network.

### Validation of the RNA-seq Data

We randomly selected four DE lncRNAs and six DE mRNAs for RT-qPCR in each comparison group, including three common differentially expressed lncRNAs and two common differentially expressed mRNAs. Primers (Supplementary Table 1) were designed using Primer 5.0 and synthesized by Sangon Biotech Co., Ltd (Shanghai, China). ACTB was selected as the internal reference. The RT-qPCR was implemented using the ChamQ SYBR qPCR Master Mix Kit (Vazyme Biotech, Nanjing, China). The relative expression was calculated with the method 2^−ΔΔCt^.

### Statistical Analysis

The significance of body weight at different embryo ages was tested using SPSS13.0 software with the method Independent-Samples *T*-Test. All data were presented as mean ± SD (standard deviation).

## Results

### Tissue Collection

We collected the body weight at 300 days of female/male chicken in parents and the body weight of offspring at 14/20-day embryo, and they were shown in Table 1. We found that the average body weight between 14-day embryos and 20-day embryos were both significant (*P* ≤ 0.05) in the two groups.

**Table 1:** Statistics of body weight for fast- and slow-growing Bian chicken.

**Slow-growing Bian chicken**		**Fast-growing Bian chicken**	
Average body weight of female chicken at 300 days (g)	Body weight of male chicken at 300 days (g)	Average body weight of female chicken at 300 days (g)	Body weight of male chicken at 300 days (g)
1,252 ± 24.73 (*n* = 12)	1,518	2,248 ± 63.11 (*n* = 12)	3,005
Average weight of 4 fertilized eggs for S14 group (g)	Average weight of 4 fertilized eggs for S20 group (g)	Average weight of 4 fertilized eggs for F14 group (g)	Average weight of 4 fertilized eggs for F20 group (g)
46.07 ± 0.86	45.89 ± 1.44	54.50 ± 0.65	56.16 ± 2.01
Average body weight of four 14-day embryos (g)	Average body weight of four 20-day embryos (g)	Average body weight of four 14-day embryos (g)	Average body weight of four 20-day embryos (g)
9.26 ± 0.55^A^	32.60 ± 1.70^B^	11.25 ± 0.63^A^	39.26 ± 2.40^B^

A, B *Different capital letters on one line in the same group indicate P ≤ 0.01*.

### Quality Control and Comparison of Sequencing Data

The quality control results of sequencing data were shown in Supplementary Table 2. Clean bases of samples have 11.95G (S14_1) at least. The percentage of the clean base with Q20 and Q30 was more than 98.00% (F20_4) and 93.99% (F20_4), respectively. The GC content of the samples ranged from 45.26 to 48.49%. All the further analyses were based on clean data with high quality.

The comparison results (Supplementary Table 3) showed that clean reads mapped to the reference genome were all more than 91.02% (F20_2). The distribution of clean reads mapped to the reference genome located in exonic, intronic and intergenic regions (Supplementary Figure 1) and the proportion of clean data mapped to exon is the highest in all samples.

### Differential Expression Analysis

**Figure 3:**
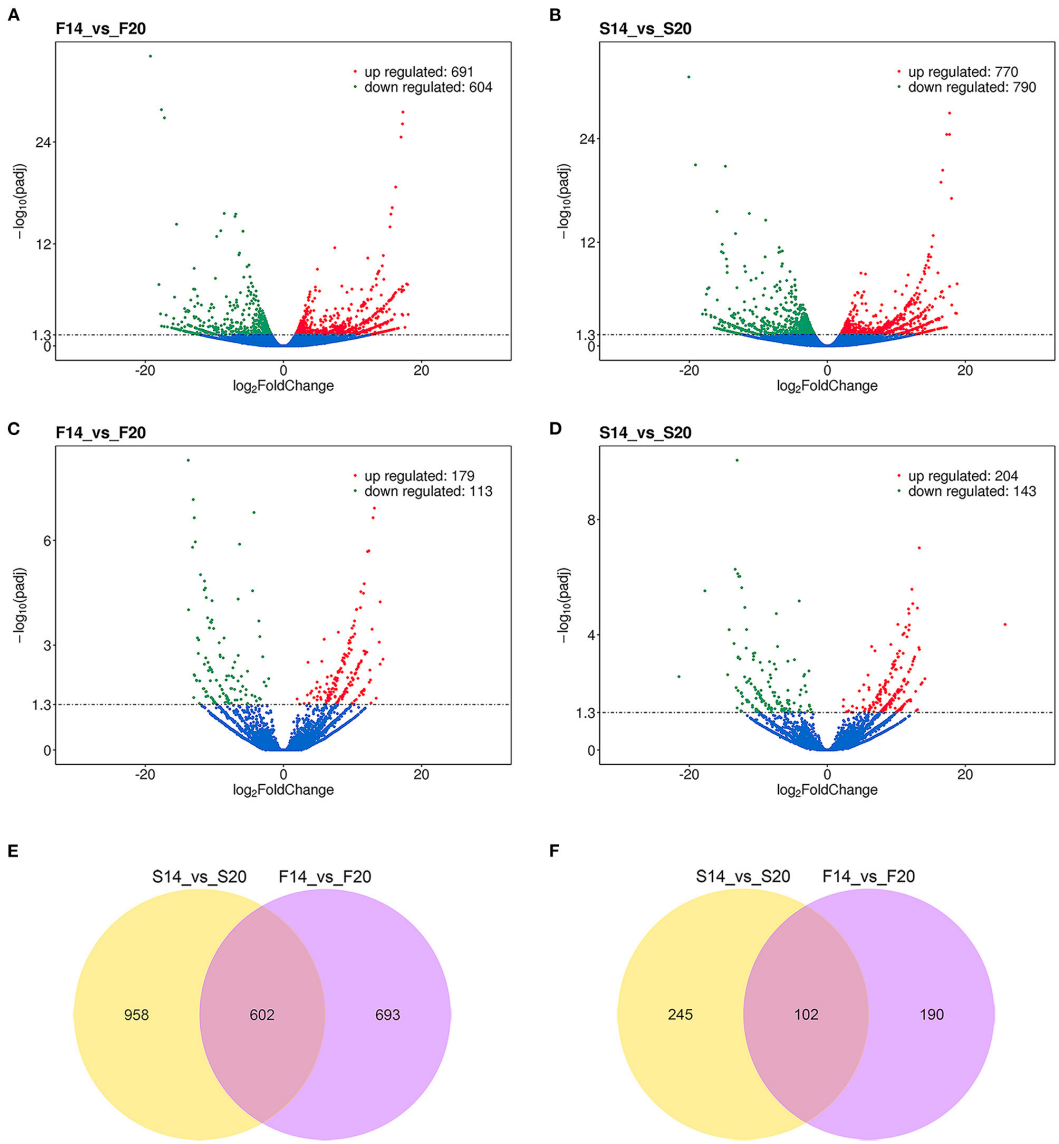
Results of differential expression analysis. **(A)** Differentially expressed mRNAs of F14vsF20; **(B)** Differentially expressed mRNAs of S14vsS20; **(C)** Differentially expressed lncRNAs of F14vsF20; **(D)** Differentially expressed lncRNAs of S14vsS20; **(E)** The same differentially expressed mRNAs in comparison groups S14vsS20 and F14vsF20; **(F)** The same differentially expressed lncRNAs in comparison groups S14vsS20 and F14vsF20.

A total of 2,253 differentially expressed (DE) mRNAs and 537 DE lncRNAs (Supplementary Table 4) were obtained with the P_adjust ≤ 0.05 in the comparison of transcriptome sequencing data of leg muscle at different embryo ages. Among them, 1,295 (Figure 3A) and 1,560 (Figure 3B) DE mRNAs were found in the comparison group F14vsF20 and S14vsS20, and 292 (Figure 3C) and 347 (Figure 3D) DE lncRNAs were identified in the two comparison groups, respectively.

**Figure 4:**
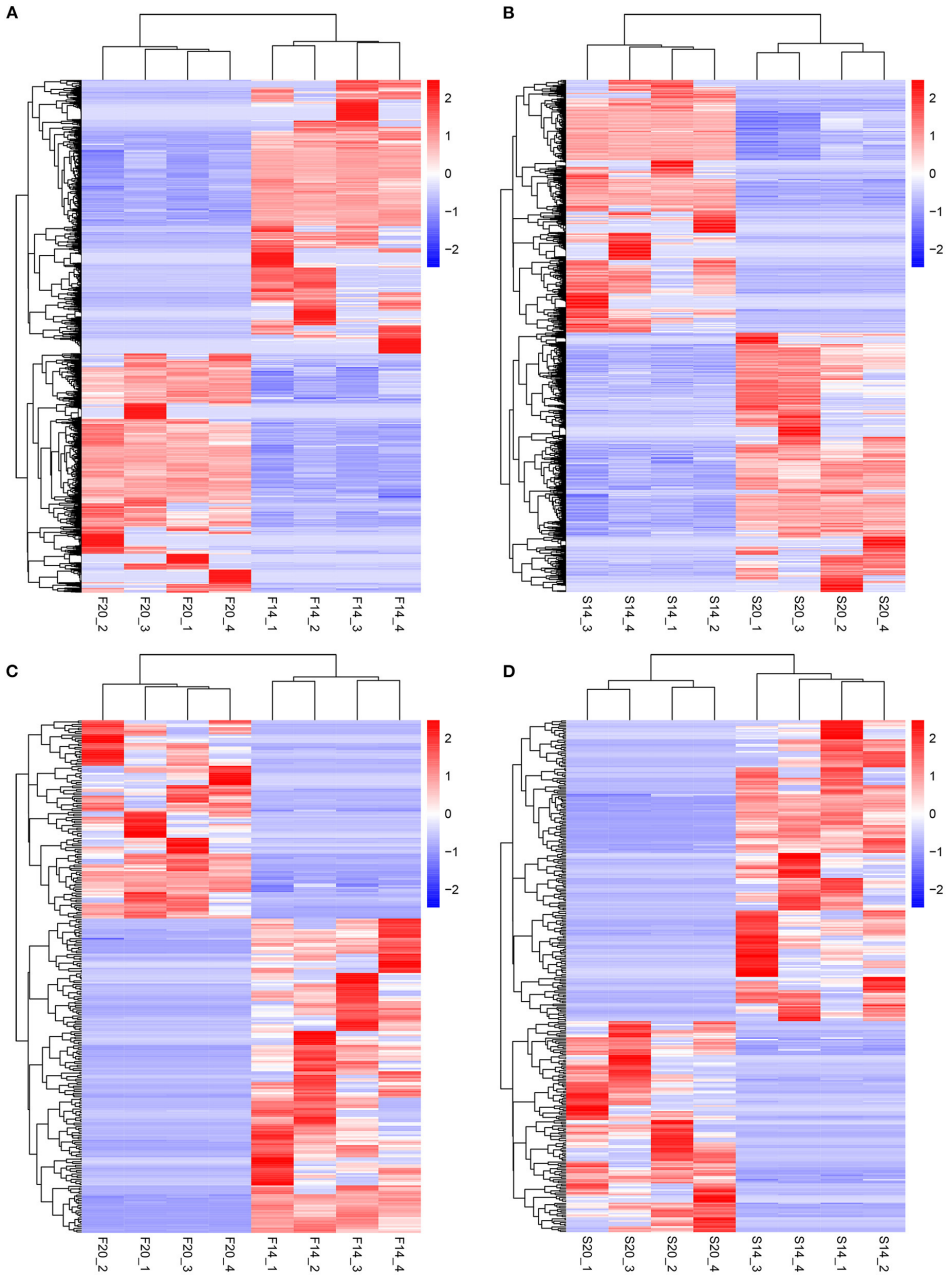
Results of hierarchical clustering analysis. **(A)** Hierarchical clustering results for mRNAs in F14vsF20; **(B)** Hierarchical clustering results for mRNAs in S14vsS20; **(C)** Hierarchical clustering results for lncRNAs in F14vsF20; **(D)** Hierarchical clustering results for lncRNAs in S14vsS20.

Further analysis showed that there were 602 same DE mRNAs (Figure 3E) and 102 (Figure 3F) same DE lncRNAs between the two comparison groups, F14vsF20 and S14vsS20. Finally, hierarchical clustering analysis was performed with the expression of DE mRNAs (Figures 4A,B) and DE lncRNAs (Figures 4C,D), and it showed that samples in the same group were well-clustered together.

### Functional Enrichment Analysis for the Target DE mRNAs of DE lncRNAs

**Figure 5:**
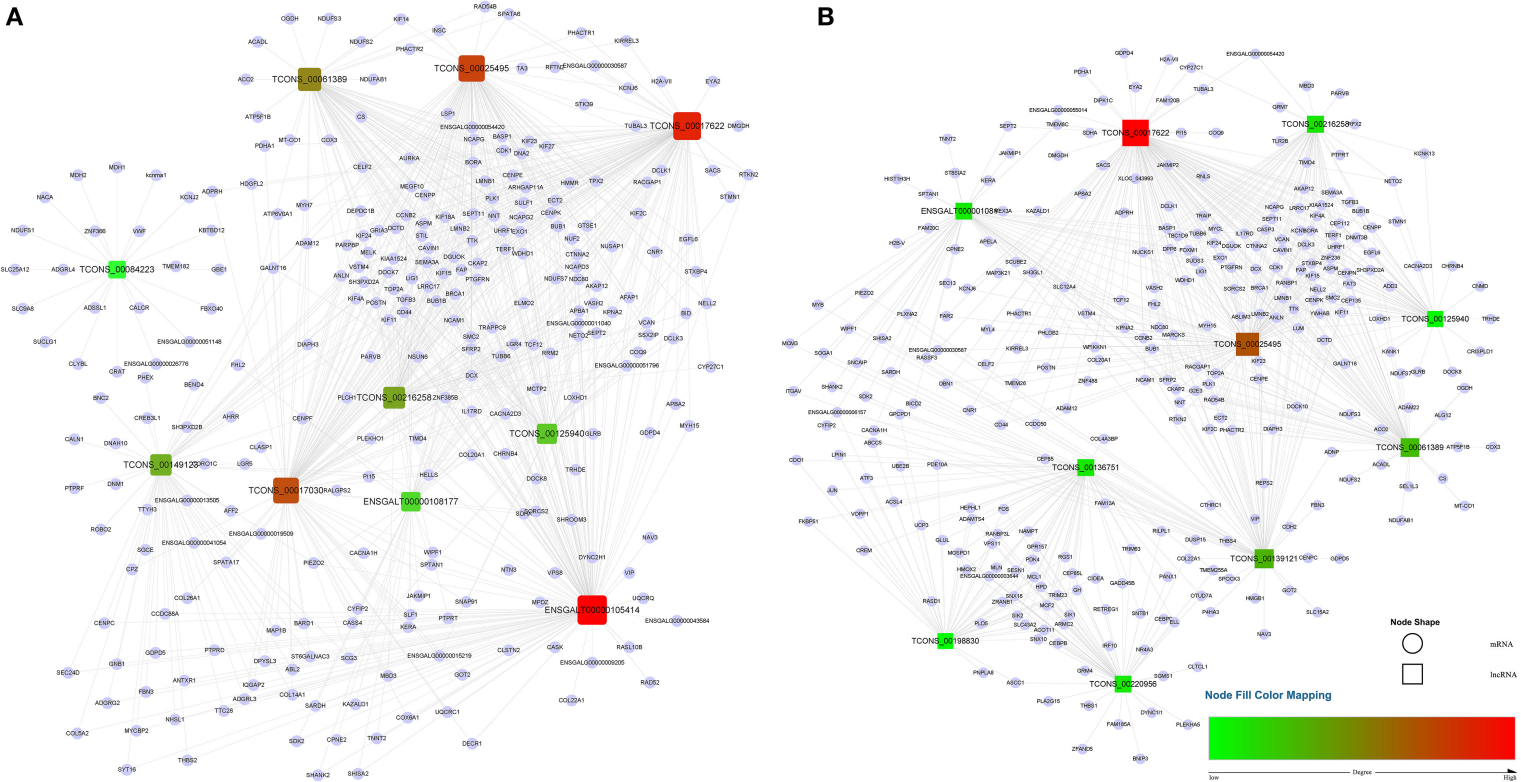
lncRNA-mRNA co-expression networks for the top 10 lncRNAs. **(A)** lncRNA-mRNA network of F14vsF20; **(B)** lncRNA-mRNA network of S14vsS20. Red indicates high degree and green indicates low degree.

**Figure 6:**
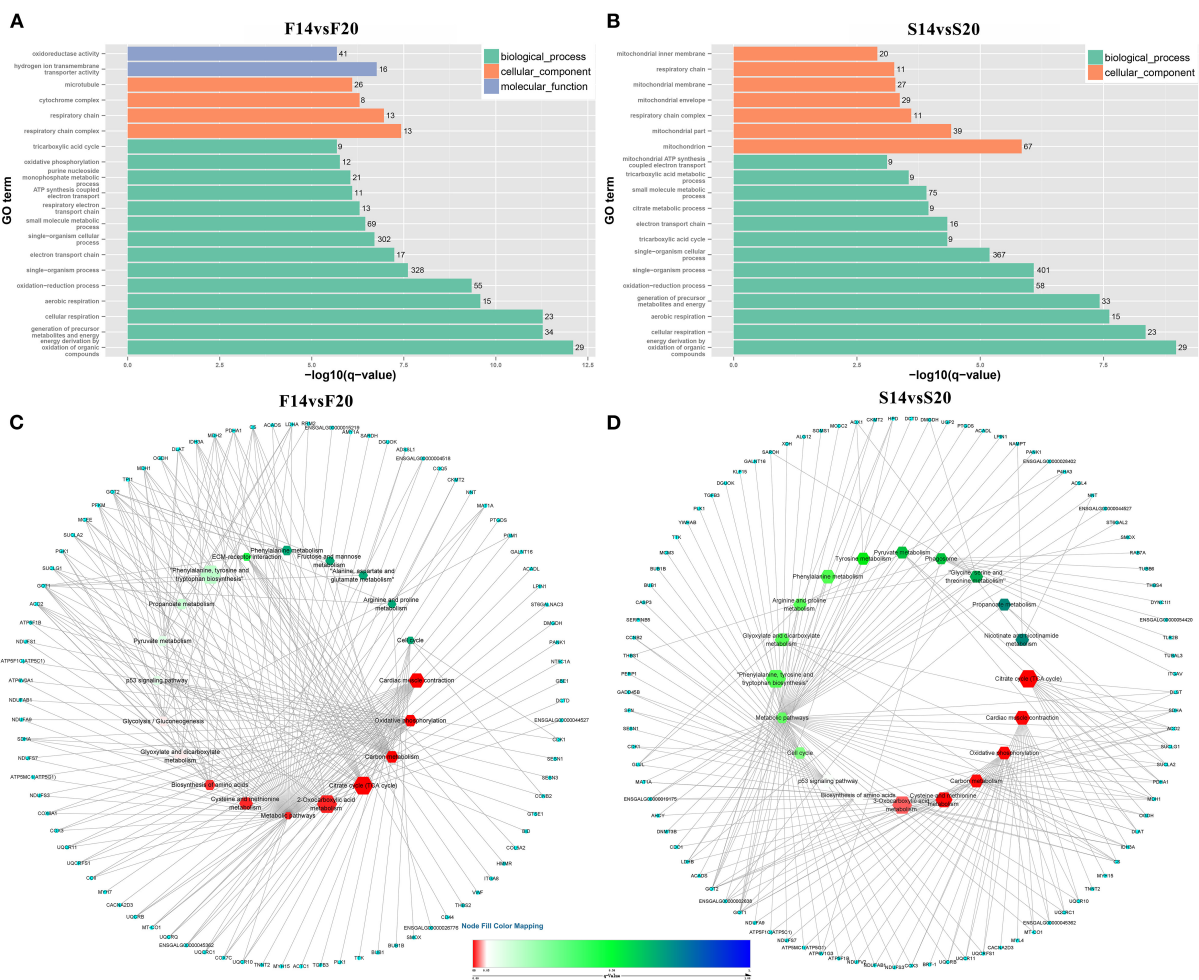
Functional enrichment analysis of targeted DE mRNAs **(A)** GO enrichment analysis for targeted DE mRNAs of F14vsF20; **(B)** GO enrichment analysis for targeted DE mRNAs of S14vsS20; **(C)** KEGG pathway enrichment for targeted DE mRNAs of F14vsF20; **(D)** KEGG pathway enrichment for targeted DE mRNAs of S14vsS20. **(C,D)** The hexagon of the inner ring represents the pathways, and the circle of the outer ring represents the enriched genes.

A total of 1,029 co-expression pairs were predicted between DE lncRNAs and DE mRNAs in the comparison group F14vsF20. The first 10 lncRNAs, including ENSGALT00000105414, TCONS_00017622, TCONS_00025495, etc., were displayed according to the degree in Figure 5A (Supplementary Table 5). GO analysis for the targeted DE mRNAs of total DE lncRNAs showed that 166 terms with P_adjust ≤ 0.05 were obtained (Supplementary Table 6). Most of the top 20 GO terms were related to energy metabolism, such as energy derivation by oxidation of organic compounds, generation of precursor metabolites and energy, cellular respiration, etc. (Figure 6A). KEGG enrichment analysis showed that the first 20 pathways were all related to growth and development, and most of the significantly enriched 10 pathways (P_adjust ≤ 0.05) were involved in energy metabolism, such as oxidative phosphorylation, carbon metabolism, citrate cycle (TCA cycle), etc. (Figure 6C). It is speculated that the growth of leg muscle tissue in the embryonic stage is active and needs a lot of energy.

In group S14vsS20, 1,427 lncRNA-mRNA pairs were identified for the DE lncRNAs and DE mRNAs. From Figure 5B (Supplementary Table 5), we could find that 6 of the top 10 lncRNAs sorted by degree are the same as that of group F14vsF20, which suggested that these lncRNAs were important in both fast and slow-growing chickens. A total of 64 significantly enriched GO terms (Supplementary Table 7) were obtained for these DE mRNAs. Compared with group F14vsF20, the same GO items were up to 12 in the top 20 terms, and most of them were also related to energy metabolism, such as energy derivation by oxidation of organic compounds, cellular respiration, aerobic respiration, oxidation-reduction process, etc. (Figures 6A,B). KEGG enrichment showed that up to 16 pathways were the same as that of F14vsF20 and most of them were related to growth and development, such as citrate cycle (TCA cycle), cardiac muscle contraction, oxidative phosphorylation, carbon metabolism, etc. And some of them were also involved in energy metabolism, including the citrate cycle (TCA cycle), oxidative phosphorylation and carbon metabolism (Figure 6D).

### Construction of Candidate Gene Network and Theirs Function Analysis

**Figure 7:**
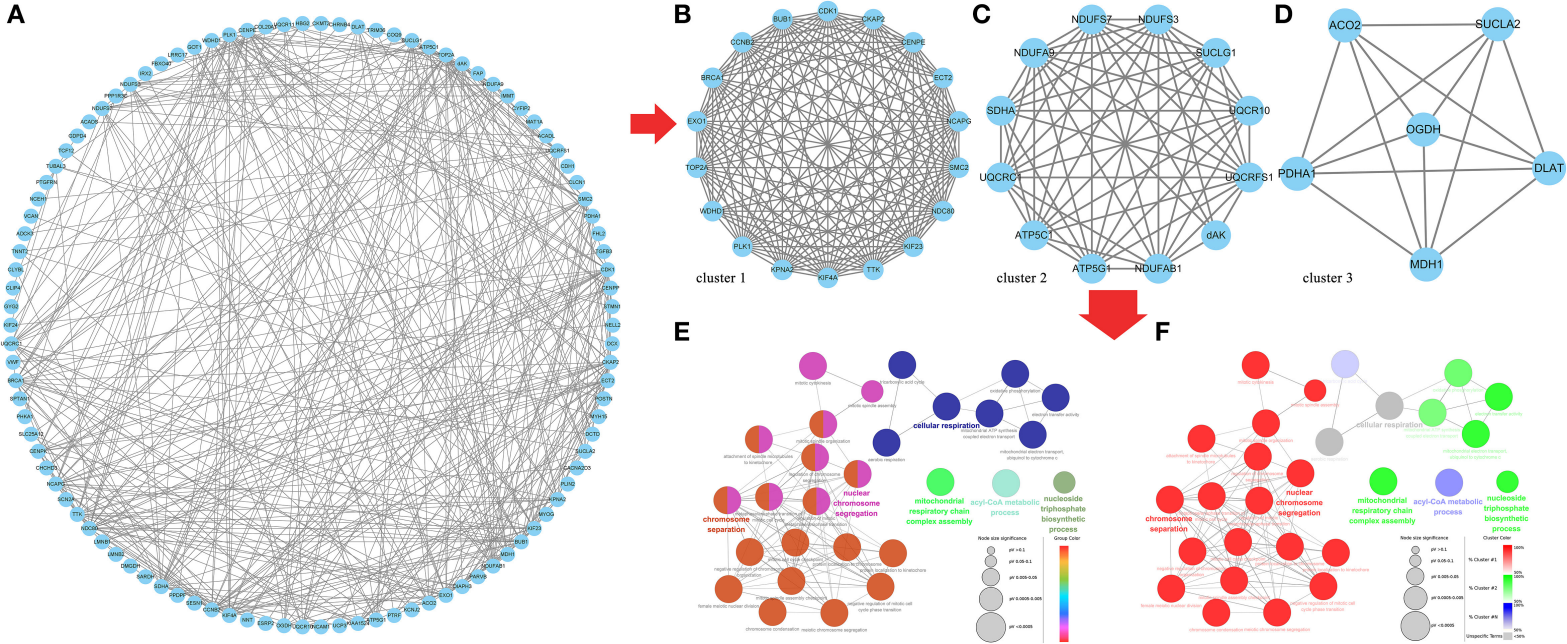
Core genes identification and functional analysis for the same targeted DE genes in F14vsF20 and S14vsS20. **(A)** Protein-protein interaction network for the same targeted DE genes; **(B)** The core network 1(cluster 1) analyzed by MCODE; **(C)** The core network 2 (cluster 2) analyzed by MCODE; **(D)** The core network 3 (cluster 3) analyzed by MCODE; **(E)** Functional analysis of biological processes for the genes in the three core networks; **(F)** Functional analysis of biological processes for the genes in each core network.

Finally, we predicted the target relationship between 602 same DE mRNAs and 102 same DE lncRNAs in the two comparison groups, F14vsF20 and S14vsS20, and found 442 lncRNA-mRNA pairs (Supplementary Table 8), including 201 mRNAs and 32 lncRNAs. And then we constructed the PPI regulatory relationship for the 201 targeted mRNAs with the STRING database and visualized the network in Figure 7A. Three core networks (cluster 1, cluster 2 and cluster 3) were then obtained using the plug-in MCODE of Cytoscape (Figures 7B–D).

And the plug-in ClueGo was used to further analyze the function of genes in all clusters (Figure 7F). Results showed that these genes were mainly enriched in six kinds of biological processes (Figure 7E) and the representative terms of the six groups were nuclear chromosome segregation, chromosome separation, mitochondrial respiratory chain complex assembly, nucleoside triphosphate biosynthetic process, cyl-CoA metabolic process and cellular respiration, respectively (Figure 7E). Among them, the genes in cluster 1 were mainly involved in the biological processes represented by nuclear chromosome segregation and chromosome separation, genes in cluster 2 were mainly related to the biological processes of mitochondrial respiratory chain complex assembly and nucleoside triphosphate biosynthetic process and genes in Cluster 3 participated in cyl-CoA metabolic process. In addition, the biological process represented by cellular respiration was enriched by the genes of all the three clusters (Figure 7F).

### Validation of RNA-seq Results Using RT-qPCR

**Figure 8:**
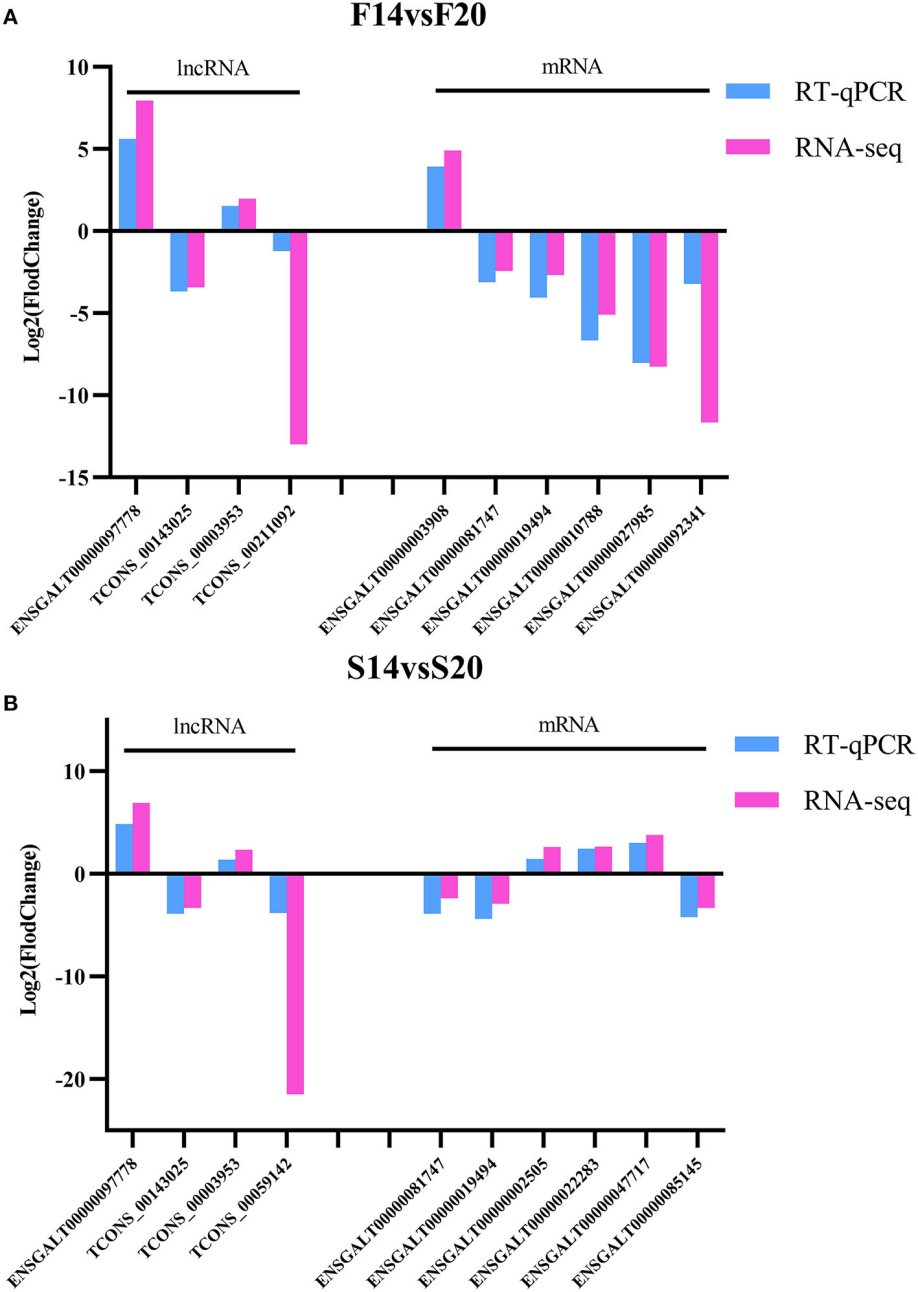
Validation for the results of RNA-seq. **(A)** DE lncRNAs and mRNAs for F14vsF20 comparison group; **(B)** DE lncRNAs and mRNAs for S14vsS20 comparison group.

Four DE lncRNAs and six DE mRNAs were randomly selected for RT-qPCR to verify the accuracy of RNA-seq in each comparison group. LncRNAs contain TCONS_00059142, ENSGALT00000097778, TCONS_00143025, TCONS_00003953 and TCONS_00211092. MRNAs include ENSGALT00000003908, ENSGALT00000081747, ENSGALT00000019494, ENSGALT00000010788, ENSGALT00000027985, ENSGALT00000092341, ENSGALT00000002505, ENSGALT00000022283, ENSGALT00000047717 and ENSGALT00000085145. The results (Figure 8) showed that expression trends of the DE lncRNAs and mRNAs between different groups in RT-qPCR were consistent with that of RNA-seq, which further showed that the sequencing data is accurate and reliable.

## Discussion

Chicken muscle is an important source of animal protein for humans, and the growth and development of muscle, especially skeletal muscle, directly determines the quality and quantity of poultry meat, further affecting the economic benefits. LncRNA widely exists in eukaryotic organisms and it can function through diverse mechanisms of action (30). It could modulate transcription, epigenetic modifications, protein/RNA stability, translation, and posttranslational modifications by interacting with DNA, RNAs and/or proteins (31).

In the study, we collected leg muscles of Bian chicken at 14-day and 20-day embryo ages for RNA-seq. By constructing the lncRNA-mRNA pairs between DE lncRNAs and DE mRNAs with co-expression relations, six lncRNAs in the top 10 were found to be consistent between the fast- and slow-growing groups, including ENSGALT00000108177, TCONS_00125940, TCONS_00216258, TCONS_00061389, TCONS_00025495, TCONS_00017622. Functional enrichment analysis showed that 12 of the first 20 GO terms were the same between the fast- and the slow-growing groups. And 16 of the first 20 KEGG pathways were also the same in the fast- and slow-growing groups. Among them, the citrate cycle (TCA cycle), oxidative phosphorylation and carbon metabolism were closely related to respiration. In addition, the same pathways, biosynthesis of amino acids, p53 signaling pathway and cell cycle, were vital to life activities. All the above results showed that the growth and development of skeletal muscle were highly similar in the fast- and slow-growing groups.

We further explored the same targeted DE mRNAs between the two comparison groups, F14vsF20 and S14vsS20, and 201 mRNAs were finally found. The database String (11.5) is used to predict the interaction between them and three core networks (cluster 1, cluster 2 and cluster 3) were obtained using Cytoscape. To further understand the function of genes in the three core networks, we used the plug-in ClueGo to analyze the biological process of these mRNAs.

Genes of cluster 1 are mainly involved in biological processes such as nuclear chromosome segregation and chromosome separation, and the enriched genes are BUB1, CENPE, ECT2, KIF23, KIF4A, NCAPG, NDC80, PLK1, SMC2, TOP2A and TTK. The co-differentially expressed lncRNAs with these genes were TCONS_00061389, TCONS_00025495, TCONS_00017622, ENSGALT00000108177, TCONS_00216258 and TCONS_00125940. From Figure 5, we can find that all these lncRNAs are at the top10 in both of the fast- and slow-growing comparison groups. Combined with the KEGG pathway results (Figure 6), we analyzed that only three genes PLK1, BUB1 and TTK were enriched in the top20 pathway, and all of them were enriched in the cell cycle pathway. TCONS_00061389, TCONS_00025495, and TCONS_00017622 have co-expression target relationships with PLK1, BUB1 and TTK. PLK1 is known as a major regulator of the metaphase to anaphase transition of division (32). BUB1 is a conserved kinase critical for the spindle assembly checkpoint and it also facilitates chromosome alignment and contributes to the regulation of mitotic duration (33). TTK (also known as Mps1) is the core component of the spindle assembly checkpoint, which ensures the proper distribution of chromosomes to daughter cells to maintain genome integrity and to balance growth and division (34). During myogenesis, myoblasts proliferate and differentiate in large numbers to complete muscle development, and this process is strongly regulated by these genes and theirs co-differentially expressed lncRNAs.

Genes of cluster 2 are mainly involved in the biological processes of nucleoside triphosphate biosynthetic process and mitochondrial respiratory chain complex assembly. There are three genes [ATP5C1 (ATP5F1C), ATP5G1 (ATP5MC1), SUCLG1] in the nucleoside triphosphate biosynthetic process. ATP5C1 and ATP5G1 are one of the subunits of ATP synthase (35, 36), which is responsible for the generation of ATP through phosphorylation of ADP by using electrochemical energy generated by the proton gradient across the inner membrane of mitochondria (37). KEGG pathway analysis (Figure 6) showed that ATP5C1 and ATP5G1 were enriched in oxidative phosphorylation and metabolic pathways, which were both in the top 20. SUCLG1 was enriched in four pathways, which were carbon metabolism, citrate cycle (TCA cycle), metabolic pathways and propanoate metabolism. Succinate-CoA ligase (SUCL) is a heterodimer consisting of an alpha subunit encoded by SUCLG1, and a beta subunit encoded by either SUCLA2 or SUCLG2 catalyzing an ATP- or GTP-forming reaction, respectively, in the mitochondrial matrix (38, 39). At the same time, among the top 10 lncRNAs of the lncRNA-mRNA network of F14vsF20 (Figure 4A), lncRNA TCONS_00084223 targeting SUCLG1 was found.

Four genes were enriched in another biological process, mitochondrial respiratory chain complex assembly, and they were NDUFA9, NDUFAB1, NDUFS7 and UQCR10. A series of large membrane protein complexes in the inner mitochondrial membrane (IMM) is responsible for the assembly, including complex I, complex II, complex III, complex IV and ATP synthase (40). The three genes NDUFA9, NDUFAB1, and NDUFS7 belong to the subunit of complex I (40). Pathway enrichment analysis showed that NDUFA9, NDUFAB1, and NDUFS7 were enriched in oxidative phosphorylation and metabolic pathways, and both the two pathways were in the top 20 (Figure 6). Combined with the lncRNA- mRNA regulatory network (Figure 4), we can find that both NDUFAB1 and NDUFS7 have a targeting relationship with lncRNA TCONS_00061389, which is also in the first 10 lncRNAs of S14vsS20. UQCR10 is an important component of complex III (41). It was enriched in three pathways cardiac muscle contraction, oxidative phosphorylation and metabolic pathways (Figure 6). Mitochondria are the power plants of the eukaryotic cell and they produce most of the huge amounts of ATP needed for the functioning of the cell (40). These mRNAs and lncRNAs related to mitochondrial respiration play an important role in chicken growth and development.

The genes in cluster 3 are mainly enriched in the biological process represented by the acyl-CoA metabolic process and the enriched genes are DLAT, PDHA1, and SUCLA2. DLAT and PDHA1 are one of the subunits of the mitochondrial pyruvate dehydrogenase complex (42, 43), which occupied a key position in the oxidation of glucose by linking the glycolytic pathway to the oxidative pathway of the tricarboxylic acid cycle (44). Pathway enrichment analysis showed that in the top 20 pathways, the two genes were enriched in the same four pathways, the citrate cycle (TCA cycle), carbon metabolism, metabolic pathways and pyruvate metabolism. In the lncRNA-mRNA network, TCONS_00017622 targeting to PDHA1 were found in the first 10 lncRNA (Figure 5). The function of SUCLA2 has been mentioned above together with SUCLG1 and it was enriched in four of the top 20 pathways, which are carbon metabolism, citrate cycle (TCA cycle), metabolic pathways and propanoate metabolism.

Finally, the genes enriched in item cellular respiration come from the three clusters and they are NDUFS7, SUCLG1, SUCLA2, UQCR10, UQCRC1, UQCRFS1, CDK1, SDHA, ACO2, and MDH1. The first four genes have been introduced above. UQCRC1 and UQCRFS1 are one of the subunits of complex III (41) in the inner mitochondrial membrane, and their enrichment pathways were also the same as that of UQCR10. CDK1 is a kind of cyclin-dependent kinases (CDKs), which are serine/threonine kinases whose activity depends on a regulatory subunit—a cyclin. Once cells have duplicated their DNA, CDK1 becomes activated by A- and B-type cyclins, promoting cellular processes such as centrosome maturation and separation, chromosome condensation and mitotic entry after nuclear envelope breakdown (45). CDK1 gene is enriched in two of the top 20 pathways, the p53 signaling pathway and cell cycle. Among the first 10 lncRNAs (Figure 5), there are 4 lncRNAs with target relationship, which are TCONS_00216258, TCONS_00061389, TCONS_00025495 and TCONS_00017622, respectively.

SDHA is a subunit of succinate dehydrogenase (SDH), which is a mitochondrial enzyme involved both in the tricarboxylic acid cycle and electron transport chain (46). ACO2 is a mitochondrial protein and it could catalyze the conversion of citrate to isocitrate within the tricarboxylic acid cycle (TCA) (47). MDH1 participated in the malate/aspartate shuttle, which would regulate the tricarboxylic acid cycle in mitochondria (48). The above three genes are closely related to oxidative phosphate. Pathway analysis showed that they were enriched in Carbon metabolism, citrate cycle (TCA cycle), metabolic pathways in the first 20 pathways (Figure 6). These pathways play an important role in life activities. Co-expression analysis found that three lncRNAs TCONS_00017622, TCONS_00061389 and TCONS_00084223 in the first 10 targeted to SDHA, ACO2, and MDH1, respectively, and regulated cell life activities together.

## Conclusions

In the study, RNA-seq of leg muscle at different embryonic stages of Bian chicken showed that the embryonic development of fast-and slow-growing Bian chicken was highly similar. Several key mRNAs, lncRNAs, and lncRNA-mRNA pairs were further identified. At the same time, the important biological processes and pathways in which they are involved have also been discovered. These results would play a guiding role in the research on skeletal muscle function and also would lay a theoretical foundation for clarifying the regulatory mechanism of skeletal muscle growth and development in chicken.

## Data Availability Statement

The raw data of the study has been uploaded to the Sequence Read Archive (SRA) database and the accession number is PRJNA773377.

## Ethics Statement

The animal experiments performed in the study were all evaluated and approved by the Animal Ethics Committee of Yangzhou University.

## Author Contributions

PW performed the data analysis and drafted the manuscript. KZ, JZ, XL, and XZ contributed to laboratory experiments. PL, LZ, and QW artificially inseminated female chickens and collected eggs for hatching. TZ and KX revised the manuscript. GZ designed the study and also revised the manuscript. All authors read and approved the final manuscript.

## Funding

The study was jointly supported by the New Agricultural Breeds Creation Project in Jiangsu Province (PZCZ201730), Key R & D projects in Shanxi Province (201703D221022-3), Biological breeding Engineering (yzgc129), the China Agriculture Research System (CARS-41), and the Priority Academic Priority Academic Program Development of Jiangsu Higher Education Institutions (PAPD).

## Conflict of Interest

The authors declare that the research was conducted in the absence of any commercial or financial relationships that could be construed as a potential conflict of interest.

## Publisher's Note

All claims expressed in this article are solely those of the authors and do not necessarily represent those of their affiliated organizations, or those of the publisher, the editors and the reviewers. Any product that may be evaluated in this article, or claim that may be made by its manufacturer, is not guaranteed or endorsed by the publisher.
