# Supplementary figures and images for: Transcriptome Integration Analysis at Different Embryonic Ages Reveals Key lncRNAs and mRNAs for Chicken Skeletal Muscle
Source: Front Vet Sci. 2022 Jun 16;9:908255. doi: 10.3389/fvets.2022.908255 (PMC9244430; doi:10.3389/fvets.2022.908255)

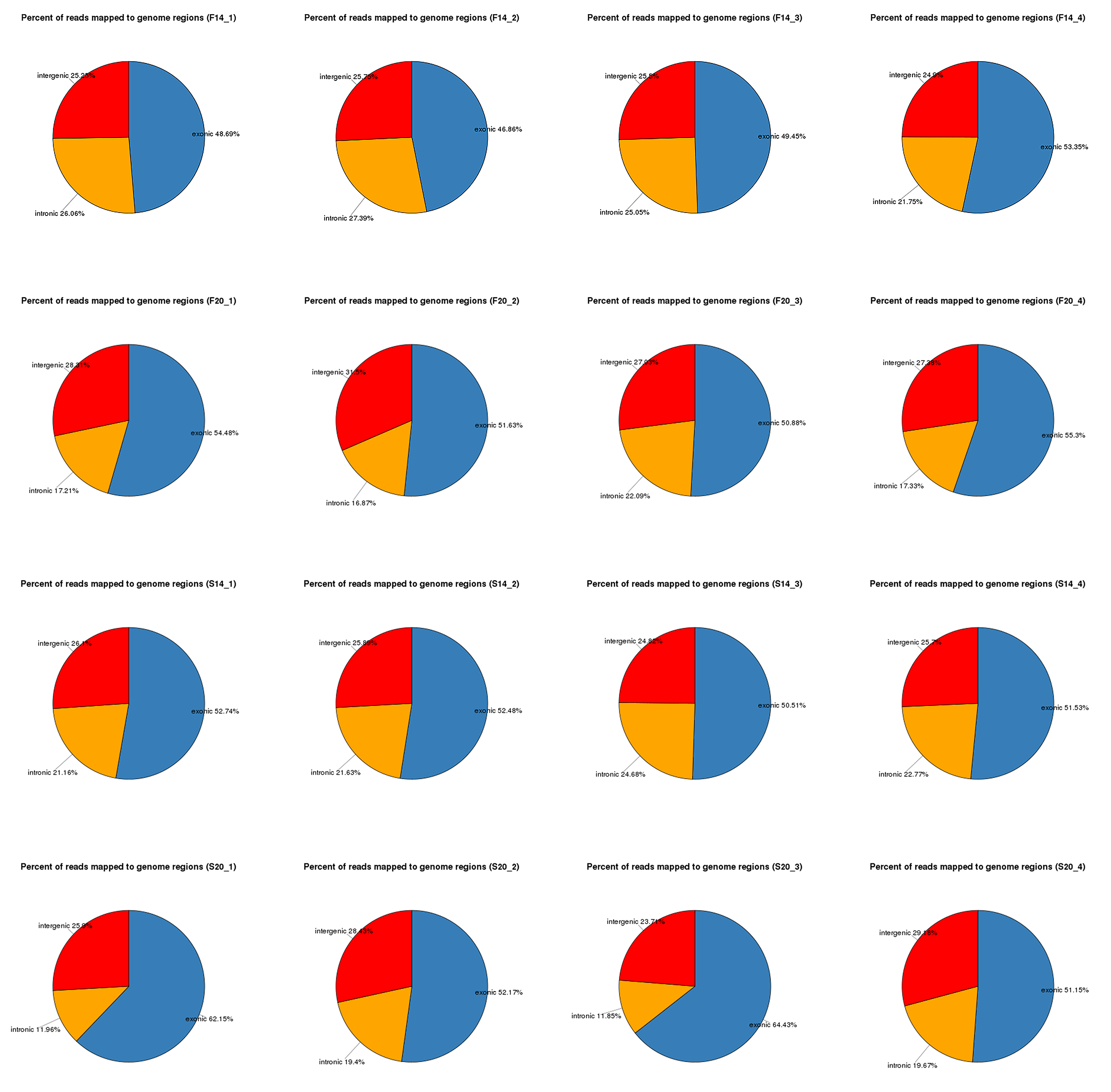

Supplement: Supplementary file 1 [file Data_Sheet_1.ZIP › Table S and Figure S/Figure S1 Percent of reads mapped to genome regions.tif]
